# Translating and Validating the Ghosting Questionnaire (GHOST) into French: Results Against the Romantic Ghosting Scale (RG-C)

**DOI:** 10.3390/ejihpe16010015

**Published:** 2026-01-18

**Authors:** Haitham Jahrami, Waqar Husain, Zahra Saif, Achraf Ammar, Khaled Trabelsi

**Affiliations:** 1 Government Hospitals, Manama 329, Bahrain; 2 Department of Psychiatry, College of Medicine and Health Sciences, Arabian Gulf University, Manama 329, Bahrain; 3 Department of Humanities, COMSATS University Islamabad, Islamabad Campus, Park Road, Islamabad 45550, Pakistan; 4 Department of Training and Movement Science, Institute of Sport Science, Johannes Gutenberg-University Mainz, 55099 Mainz, Germany; 5 Research Laboratory, Molecular Bases of Human Pathology, LR19ES13, Faculty of Medicine of Sfax, University of Sfax, Sfax 3000, Tunisia; 6 Research Laboratory Education, Motricité, Sport et Santé, EM2S, LR19JS01, High Institute of Sport and Physical Education of Sfax, University of Sfax, Sfax 3000, Tunisia; 7 Department of Movement Sciences and Sports Training, School of Sport Science, The University of Jordan, Amman 11942, Jordan

**Keywords:** communication, digital relationships, social behavior, validation

## Abstract

Background: Ghosting, the sudden cessation of communication without explanation, is a growing phenomenon in digital interactions. Objective: This study translated and validated the Ghosting Questionnaire (GHOST) into French to address the lack of a culturally appropriate tool for French-speaking populations. Methods: Using a cross-sectional design, we followed rigorous translation protocols, including forward and back translation, expert review, and pilot testing. A diverse group of 274 French-speaking adults participated in a multisite study in Bahrain and Tunisia by completing the French language GHOST and the Romantic Ghosting Scale (RG-C). Results: Psychometric analyses revealed strong reliability (Cronbach’s α = 0.880, test–retest ICC = 0.920) and a unidimensional structure (CFI = 0.963, RMSEA = 0.058). Convergent validity was confirmed through significant correlations with RG-C subscales: Role of Aggressor (r = 0.899), Role of Victim (r = 0.829), and Emotional Impact (r = 0.632, all *p* < 0.001). Item response theory analysis demonstrated proper category functioning and meaningful item hierarchy across ghosting severity levels. Conclusions: The French GHOST offers a robust instrument for researchers and clinicians to explore ghosting’s psychological impacts in French-speaking communities.

## 1. Introduction

The proliferation of digital communication platforms has significantly transformed interpersonal relationships and mental health (Husain et al., 2025). Within this context of rapid digitalization, ghosting is a behavior defined as the abrupt cessation of communication in an established relationship without explanation (Kay & Courtice, 2022). This act typically involves ignoring messages, blocking contacts, or unfriending on digital platforms (Daraj et al., 2023). Ghosting was initially recognized in the realm of romantic relationships alone (Forrai et al., 2023; Wood et al., 2023). However, this phenomenon has transcended these boundaries later and has also been utilized for infiltrating friendships, familial ties, professional domains, and even therapeutic relationships (Astleitner et al., 2023; Farber et al., 2022; Forrai et al., 2023; Freedman et al., 2022; Konings et al., 2023; Pancani et al., 2021; Powell et al., 2022; Powell et al., 2021; Timmermans et al., 2020; Wood et al., 2023). Ghosting is often driven by the desire to avoid emotional discomfort, confrontational exchanges, or interpersonal conflict (Navarro et al., 2020). The ghoster withdraws without confrontation to seek emotional relief. However, such disengagement frequently imposes psychological burdens on the ghostee. Technological advancement has normalized ghosting by providing easily accessible and anonymous modes of disengagement. Through features such as “unmatch”, “block” or “mute” communication can be severed instantaneously, fostering a culture in which interpersonal accountability is minimized (Koessler et al., 2019; Meikle, 2024). The COVID-19 pandemic further entrenched remote dating norms and reduced the perceived need for face-to-face resolution (Thomas & Dubar, 2021).

Ghosting is not restricted by medium (offline/online), relationship type (romantic, platonic, professional), or life stage (adolescents to adults) (Biolcati et al., 2021; Konings et al., 2023). Its pervasiveness and psychological consequences merit deeper investigation within scientific discourse. The most prominent conceptual framework employed to analyze ghosting is the Shannon-Weaver communication model, which outlines six core elements of communication: sender, encoder, channel, noise, decoder, and receiver (Shannon, 1949). Ghosting interrupts this chain by disabling the decoder-receiver system; the sender’s messages are received but ignored, obstructing the feedback loop and leading to perceived ambiguity and social exclusion (Jahrami et al., 2023). Additionally, psychological theories of social exclusion and rejection sensitivity provide explanatory grounding for ghostees’ emotional responses. Being ignored or “left on read” elicits pain responses such as physical injury and may precipitate chronic mistrust, especially in individuals with insecure attachment patterns or high rejection sensitivity (Freedman et al., 2024). From a personality standpoint, individuals exhibiting traits aligned with the Dark Triad (narcissism, Machiavellianism, and psychopathy) are more likely to engage in ghosting due to their lack of empathy and fear of discomfort (Jonason et al., 2021; Powell et al., 2021). In contrast, ghostees often demonstrate vulnerability, high agreeableness, or attachment anxiety, making them susceptible to psychological harm from ambiguous endings (LeFebvre & Fan, 2020). 

The psychological impact of ghosting is asymmetrical, affecting ghostees more severely. Common outcomes include confusion, depression, anxiety, low self-esteem, hopelessness, loneliness, diminished self-worth, social withdrawal, and difficulty trusting future partners (Forrai et al., 2023; Freedman et al., 2022; Thomas & Dubar, 2021; Wu & Bamishigbin, 2023). Being ghosted leads to emotional pain that arises from a sense of rejection (Freedman et al., 2022). On the other hand, ghosters may experience temporary relief. However, some report guilt, disloyalty, and a reduced sense of moral integrity (Koessler et al., 2019). In rare cases, ghosting is perceived as a necessary form of self-protection in emotionally abusive or overwhelming situations (Freedman & Powell, 2024). Despite its negative effects, some ghostees eventually reinterpret the experience as a catalyst for personal growth and enhanced emotional resilience (Thomas & Dubar, 2021).

The Shannon–Weaver communication model (Shannon, 1949) has previously been referenced to illustrate the specific point of breakdown in ghosting, particularly the intentional disabling of the decoder-receiver feedback loop, despite the presence of an intact technical channel (Jahrami et al., 2023; Daraj et al., 2023). However, we acknowledge the model’s original engineering intent and its acknowledged limitations in addressing the complexities of human interpersonal communication. Consequently, its application in this work is confined to a narrow heuristic role and is no longer utilized as the primary conceptual framework. The theoretical foundation of ghosting is now grounded in contemporary psychological models that more effectively elucidate its emotional and relational impact, including the temporal need-threat model of ostracism and social exclusion (Williams, 2007, 2009), the theory of ambiguous loss (Boss, 2016), rejection sensitivity (Downey & Feldman, 1996), and the need-to-belong/thwarted belongingness paradigm (Baumeister & Leary, 1995). Collectively, these frameworks address the reflexive pain, threats to fundamental needs (belonging, self-esteem, control, and meaningful existence), increased rejection sensitivity, and persistent uncertainty that characterize the experience of being ghosted, thereby aligning the French validation of the GHOST with current theories in interpersonal and clinical psychology.

In response to the lack of a standardized instrument to measure ghosting, Jahrami et al. (2023) developed the Ghosting Questionnaire (GHOST)—a self-report measure based on the Shannon-Weaver communication model (Jahrami et al., 2023). GHOST is the first instrument to evaluate ghosting systematically and psychometrically, intended for use in both research and clinical settings. It comprises 8 items. Each item measures key dimensions of ghosting—neglect, ambiguity, withdrawal, tardiness, inconsistency, vulnerability, absence, and relational barriers—rated on a 5-point Likert scale from 1 (never) to 5 (always). The GHOST was found highly reliable (α = 0.74, ω = 0.74, ordinal α = 0.80) and valid (CFI = 0.92, TLI = 0.90, RMSEA = 0.07, SRMR = 0.04). It has been further translated and validated in Urdu (Husain et al., 2024a), Arabic (Husain et al., 2024b), and Turkish (Özalp et al., 2025), expanding its utility across diverse linguistic and cultural groups. Furthermore, all these three translated versions, i.e., Arabic (α = 0.87, ω = 0.87; ICC = 0.89; CFI = 0.97; TLI = 0.96; RMSEA = 0.06), Turkish (α = 0.87, ω = 0.84; CFI = 0.93; TLI = 0.90; RMSEA = 0.07; SRMR = 0.04), and Urdu (α = 0.913, ω = 0.91; CFI = 0.99; TLI = 0.98; RMSEA = 0.04; SRMR = 0.01) were also found highly reliable and valid (Husain et al., 2024a, 2024b; Özalp et al., 2025). 

It is worth noting that the original English version of the GHOST was validated in a sample of adults (mean age 29.3 years, SD = 10.2) recruited internationally (Jahrami et al., 2023). Following this, the Arabic version was validated with adults (mean age 27.6 years, SD = 8.9) from Middle Eastern countries, the Urdu version was validated with Pakistani young adults and university students (mean age 22.8 years, SD = 4.1), and the Turkish version was validated with Turkish adults (mean age 28.4 years, SD = 7.6). All versions demonstrated robust psychometric properties across various cultural contexts.

Ghosting has emerged as a prevalent strategy for relationship dissolution in digitally mediated contexts, with lifetime prevalence estimates ranging from 13% to 42% across general and dating-app populations (LeFebvre et al., 2019; Timmermans et al., 2020; Freedman et al., 2019). Empirical evidence consistently demonstrates significant adverse psychological outcomes for recipients, including increased depressive symptoms, loneliness, decreased self-esteem, thwarted basic psychological needs (such as belonging, self-esteem, meaningful existence, and control), and activation of pain-related neural pathways comparable to those associated with physical ostracism (Pancani et al., 2022; Freedman et al., 2022; Forrai et al., 2023; Konings et al., 2023; Williams, 2007, 2009). This phenomenon extends beyond romantic relationships to include friendships, family ties, professional interactions, and even therapeutic relationships (Farber et al., 2022; Astleitner et al., 2023; Wood et al., 2023). The findings, replicated across North America, Europe, Latin America, and Asia, suggest that ghosting is a structural consequence of contemporary communication technologies rather than a fleeting trend (Jahrami et al., 2023; Daraj et al., 2023). The literature repeatedly calls for psychometrically robust, cross-culturally validated measurement tools (Kay & Courtice, 2022; Navarro et al., 2020; Koessler et al., 2019), highlighting the scientific necessity of developing and adapting instruments such as the GHOST.

The objective of the current study was to translate and validate the GHOST into French. Given the global prevalence of ghosting across romantic, platonic, and professional domains (Astleitner et al., 2023; Farber et al., 2022; Forrai et al., 2023; Freedman et al., 2024; Konings et al., 2023; Pancani et al., 2021; Powell et al., 2022; Powell et al., 2021; Timmermans et al., 2020; Wood et al., 2023), a French translation of the GHOST is both timely and essential. French-speaking populations across Europe, Africa, the Caribbean, and North America are intricately integrated within digital communication ecosystems (Fewou Ngouloure, 2023; Martin, 2011), rendering them equally vulnerable to the psychosocial effects of ghosting. However, to date, no standardized instrument has been available to assess ghosting experiences in these regions. The current study sought to ensure linguistic and cultural equivalence of GHOST in French populations. A culturally attuned French version of the GHOST will enable researchers and clinicians to explore the complexities of ghosting within diverse Francophone societies, where interpersonal norms, emotional expression, and conflict resolution styles may differ significantly from other societies (Adler & Graham, 2009). We also aimed to establish convergent validity by comparing French GHOST scores with those from the RG-C Romantic Ghosting Scale, hypothesizing a significant positive correlation due to shared constructs. Accordingly, the French GHOST would not only be a tool for localized assessment but also a gateway to meaningful cross-cultural comparisons.

## 2. Materials and Methods

### 2.1. Translation Process, Study Design and Reporting Guidelines

We adopted an instrumental, cross-sectional design, aligning with best practices for questionnaire development and validation. The process involved translation, cultural adaptation, and psychometric evaluation, mirroring protocols used in Arabic, Urdu, and Turkish GHOST validations. The translation process followed established international guidelines for cross-cultural adaptation of psychological instruments, specifically adhering to the principles outlined by Beaton et al. (2000) and the International Test Commission Guidelines for Translating and Adapting Tests (Gregoire, 2018). These guidelines emphasize the importance of maintaining conceptual equivalence rather than literal translation, ensuring that the translated instrument captures the same psychological constructs as the original while being culturally appropriate for the target population.

The comprehensive validation approach encompassed multiple stages of rigorous evaluation to establish the psychometric properties of the French version. This methodology aligns with contemporary standards for instrument validation and ensures that the adapted scale maintains the reliability and validity characteristics necessary for clinical and research applications in French-speaking populations.

Reporting of this validation study followed the STROBE (Strengthening the Reporting of Observational Studies in Epidemiology) guidelines to ensure transparent and comprehensive documentation of the study methodology, results, and conclusions (von Elm et al., 2008). This adherence to established reporting standards enhances the reproducibility and interpretability of the research findings.

### 2.2. Setting

Data collection occurred from April to June 2025 across Bahrain and Tunisia. In Bahrain, we utilized French-speaking population, as French is often taught as third language (besides Arabic and English). In Tunisia we used the general population, as French serves as the administrative and educational language. It is widely used in government, official documents, and the education system, facilitating communication and instruction in various academic contexts. Participants completed surveys via Google Forms, ensuring accessibility and broad geographic reach.

### 2.3. Participants, Sample Size, and Ethical Considerations

We recruited a convenience sample of 247 French-speaking adults aged 18 years or older. Inclusion criteria required self-identification as a French speaker and provision of electronic informed consent. Exclusion criteria included refusal to consent or self-reported severe mental illness. Sample size was informed by prior GHOST validations (Husain et al., 2024a, 2024b; Özalp et al., 2025) and factor analysis guidelines, recommending 5–10 participants per item. For the 8-item GHOST, a target of 80–100 participants ensured sufficient statistical power.

Participants were recruited through multiple channels including social media platforms (Facebook, Instagram), and Instant Messaging (WhatsApp, Line) lists in both Bahrain and Tunisia, community organizations, and snowball sampling. Recruitment materials described the study as examining communication experiences in relationships and included a link to the online survey hosted on Google Forms. No compensation was provided for participation.

Ethical approval for this study was granted by the Research Ethics Committee at the psychiatric Hospital, Bahrain (Code GH/PSY/REC/2025-03-018, dated 13 February 2025). All procedures were conducted in accordance with the 1964 Helsinki Declaration and its later amendments.

### 2.4. Variables

The primary outcome was the experience of being ghosted, measured by the French GHOST. Predictors included demographic factors (age, sex, relationship status). Potential confounders, such as relationship type (romantic, platonic, professional) and frequency of digital platform use, were accounted for in analyses. The RG-C Romantic Ghosting Scale served as a comparator for convergent validity, assessing ghosting behaviors and emotional impacts in romantic contexts.

### 2.5. Data Sources and Measurement

The French GHOST, an 8-item scale, evaluated ghosting experiences on a 5-point Likert scale (1 = never, 5 = always), with higher scores indicating greater ghosting exposure. Items covered neglect, tardiness, ambiguity, barriers, absence, inconsistency, vulnerability, and withdrawal. The translation process ensured linguistic and cultural fidelity through a systematic four-stage approach. The process began with forward translation, where two bilingual, native French-speaking translators independently translated the English GHOST into French, prioritizing meaning and accessibility. A consensus meeting with a research coordinator resolved any discrepancies between the translations, producing a unified French version. Following the forward translation, back translation was conducted by two new bilingual translators who were blind to the original English version. These translators converted the French version back into English to verify the accuracy of the translation and ensure that the meaning had been preserved throughout the process. An expert review panel consisting of psychologists, psychometricians, and French linguists then evaluated both versions of the instrument. This panel assessed the translations for linguistic accuracy, cultural appropriateness, and conceptual equivalence between the original and translated versions. Based on their evaluation, refinements were made to address any identified issues.

The final stage involved pilot testing with a sample of 30 native French speakers who tested the provisional French GHOST. These participants provided valuable feedback on comprehension and clarity, which informed minor revisions that finalized the scale and ensured its effectiveness for French-speaking populations.

The RG-C, a three-factor scale, measured romantic ghosting, including behaviors (e.g., blocking profiles, ignoring messages) and emotional impacts (e.g., sadness, confusion). Both scales were administered online, with mandatory fields to eliminate missing data.

### 2.6. Descriptions of the Measures

#### 2.6.1. GQ/GHOST

The French GHOST was based on the original English language GHOST which was developed by Jahrami et al. (2023) to provide the first self-reported measure for assessing the experience of being ghosted. Initially designed with 10 items, the questionnaire was refined through rigorous item reduction analysis, resulting in a final validated version consisting of eight items. These items are designed to evaluate various aspects of ghosting, including neglect, tardiness, ambiguity, barriers, absence, inconsistency, vulnerability, and withdrawal. Participants respond to each item on a 5-point Likert scale, ranging from 1 (never) to 5 (always), with higher scores indicating a greater frequency of experiencing ghosting behaviors. In its original English version, the GHOST demonstrated adequate reliability and validity, with a Cronbach’s alpha (α) of 0.74, a McDonald’s omega (ω) of 0.74, and an ordinal alpha of 0.80. The questionnaire is designed to be completed quickly, typically taking less than 5 min. Beyond its original development, the GHOST has been successfully translated and validated in several other languages, further confirming its robust psychometric properties across diverse cultural contexts. For instance, the Arabic version (Husain et al., 2024b) showed high reliability (α = 0.87, ω = 0.87) and strong test–retest reliability (ICC 0.89), the Urdu version (Husain et al., 2024a) reported excellent internal consistency (α = 0.913, ω = 0.915) and high test–retest reliability (ICC = 0.960), and the Turkish adaptation (Özalp et al., 2025) also showed high reliability (α = 0.876–0.879, ω = 0.884–0.886). GHOST takes about five minutes to complete. 

The final approved translation is: GHOST Item 1 original English is = Did you get stood up or had your plans canceled by them without being told beforehand; French translation = Ont-ils annulé un rendez-vous ou une sortie sans vous prévenir à l’avance. GHOST Item 2 original English is = Their reply/response messages are delayed; French translation = Leurs messages de réponse arrivent avec du retard. GHOST Item 3 original English is = Their reply/response messages are confusing and vague; French translation = Leurs messages de réponse sont confus et vagues. GHOST Item 4 original English is = Have you been blocked or deleted from their social media apps or messaging apps; French translation = Vous ont-ils bloqué(e) ou supprimé(e) de leurs réseaux sociaux ou applications de messagerie. GHOST Item 5 original English is = The phrase “I’m busy” is always used in their communications; French translation = L’expression «je suis occupé(e)» est toujours utilisée dans leurs messages. GHOST Item 6 original English is = Their interest in you is inconsistent sometimes very engaged, sometimes completely uninterested; French translation = Leur intérêt pour vous est instable, parfois très engagé, parfois totalement absent. GHOST Item 7 original English is = They don’t share personal information about themselves with you; French translation = Ils ne partagent pas d’informations personnelles à leur sujet avec vous. GHOST Item 8 original English is = They are not interested in meeting; French translation = Ils ne montrent pas d’intérêt à vous rencontrer.

#### 2.6.2. Romantic Ghosting Scale (RG-C)

The RG-C was developed by Herrera-López and colleagues (Herrera-López et al., 2024) to analyze the psychometric properties of a ghosting scale specifically designed for romantic relationships within a Colombian sample. This instrumental study aimed to address a notable gap in research concerning ghosting in the Latin American context, delineating the roles of involvement (aggressor and victim) and the emotional impact of the phenomenon, rooted in the theory of ghosting and adapted to relational violence. The scale’s development involved content validity assessment by experts and a pilot test, resulting in an initial scale of 18 Likert-type items. After rigorous exploratory factor analysis (EFA), 3 items were rejected due to communalities or saturations below thresholds, leading to a final validated version composed of 15 global items. These items are structured into three distinct factors: “Role of the Aggressor”, “Role of the Victim”, and “Emotional Impact”. The first 10 items, which assess the aggressor and victim roles, utilize a unique dual response system, asking participants “How often does/did the person use this behavior against you?” and “How often do/did you use this behavior against them?”. All items are rated on a 5-point Likert scale.

The RG-C demonstrated excellent internal consistency, with Cronbach’s alpha (α) coefficients of 0.90 for Role of Victim, 0.86 for Role of Aggressor, and 0.87 for Emotional Impact. McDonald’s omega (ω) coefficients were similarly high: 0.91 for Role of Victim, 0.88 for Role of Aggressor, and 0.88 for Emotional Impact. Composite reliability (CR) indices were also reported at 0.89 for all three factors. Content validity was high, with a V-Aiken Total of 0.88. Construct validity was confirmed through both exploratory factor analysis (EFA) and Confirmatory Factor Analysis (CFA). The CFA results showed optimal model fits, including a Comparative Fit Index (CFI) of 0.992, a Non-Normality Fit Index (NNFI) of 0.990, and a Root Mean Square Error of Approximation (RMSEA) of 0.042. The explained variance from the EFA was 47.02%. RG-C takes about five minutes to complete.

The RG-C (Herrera-López et al., 2024) was chosen as the criterion for convergent validity for the French GHOST due to its thorough evaluation of ghosting behaviors and their emotional impacts within romantic relationships. The RG-C consists of 15 items organized into three theoretically distinct subscales that conceptually align with the GHOST framework. Role of Aggressor (5 items): This subscale assesses ghosting behaviors enacted by the respondent, directly paralleling GHOST’s evaluation of withdrawal, avoidance, and communication barriers. Role of Victim (5 items): This subscale measures ghosting behaviors experienced by the respondent, employing similar wording to that of the Aggressor subscale. This bidirectional assessment is particularly valuable, as the GHOST primarily captures the victim perspective, making the RG-C Victim subscale the closest theoretical match. Emotional Impact (5 items): This subscale evaluates the psychological and emotional consequences of being ghosted, capturing dimensions of ambiguity, vulnerability, and emotional distress that are central to the GHOST construct.

### 2.7. Bias

To minimize translation bias, we adhered to cross-cultural adaptation guidelines. Sampling bias was reduced by recruiting from varied regions and platforms. Non-response bias was addressed through mandatory survey fields and clear consent procedures. Self-selection bias was acknowledged as a potential limitation. The RG-C was administered in original language format (English) to maintain its psychometric properties. 

### 2.8. Quantitative Variables

Continuous variables (e.g., GHOST and RG-C scores) were summarized as means and standard deviations. Categorical variables (e.g., sex, relationship type) were grouped for subgroup analyses. Likert responses were treated as interval data.

### 2.9. Statistical Methods

Analyses were conducted using R software packages, with a significance threshold of *p* ≤ 0.05 established for all statistical tests. Specifically, we used lavaan package (Rosseel, 2012) for CFA, the mirt package (Chalmers, 2012) for item response theory analysis, the psych package (Revelle, 2025) for calculating reliability measures such as Cronbach’s alpha and McDonald’s omega, the eRm package (Mair et al., 2025) for polytomous Rasch modeling, the corrplot package (Wei et al., 2024) for Pearson correlation analyses, and the semTools package (Jorgensen et al., 2025) for measurement invariance testing. The analytical approach encompassed multiple dimensions of psychometric evaluation to ensure the robustness and validity of the French GHOST instrument.

Reliability was assessed through multiple measures to evaluate the internal consistency and temporal stability of the scale. Cronbach’s alpha and McDonald’s omega coefficients were calculated to assess internal consistency, with a target threshold of ≥0.70 considered acceptable. Test–retest reliability was evaluated in a subsample of 50 participants after a two-week interval using the Intraclass Correlation Coefficient (ICC), with a target value of >0.80 indicating good temporal stability.

Construct validity was examined through CFA using maximum likelihood estimation to test the unidimensional structure of the instrument. Multiple fit indices were employed to evaluate model adequacy, including the Comparative Fit Index (CFI), Tucker–Lewis Index (TLI), and Normed Fit Index (NFI), with values ≥0.90 considered indicative of good fit. Additionally, the Root Mean Square Error of Approximation (RMSEA) and Standardized Root Mean Square Residual (SRMR) were evaluated, with values ≤0.08 considered acceptable. Factor loadings ≥0.4 were deemed satisfactory for item retention.

Measurement invariance was tested across groups using multi-group confirmatory factor analysis (MG-CFA). The analyses examined configural, metric, and scalar invariance, with changes in CFI (∆CFI) ≤ 0.01 indicating measurement equivalence between groups. Measurement invariance analyses were conducted based on country, sex, and relationship status.

Item Response Theory (IRT) analysis was conducted using a graded response model to assess individual item characteristics. This analysis evaluated item difficulty and discrimination parameters, providing detailed information about item performance and informing decisions about the instrument’s effectiveness across different levels of the measured construct.

Convergent validity was examined through Pearson’s correlation analyses to investigate the associations between French GHOST scores and scores on the RG-C measure. The analysis was based on the hypothesis that these measures would demonstrate a significant positive relationship, supporting the convergent validity of the French GHOST.

All analyses were adjusted for potential confounding variables, including age, relationship type, and platform use patterns. Missing data were not a concern in this study due to the implementation of mandatory response fields in the data collection protocol.

## 3. Results

### 3.1. Participants

The sample comprised 76.3% females (N = 209) and 23.7% males (N = 65), with a mean age of 23.6 years (SD = 4.07). Additionally, 63.9% of participants reported that they are currently in or have ever been in a romantic or marital relationship in the past six months (N = 175), while 36.1% indicated that they have not (N = 99). It is important to note that the GHOST assesses ghosting experiences across all relationship types—romantic, platonic, familial, and professional—not exclusively romantic relationships. Therefore, participants without recent romantic relationship experience could still report ghosting experiences in friendships, family relationships, workplace contexts, or past romantic relationships beyond the six-month timeframe (Table 1).

### 3.2. Outcome Data

#### 3.2.1. GQ Results

Individual GHOST items showed varying response patterns. GHOST#2 had the highest mean score (2.52, SD = 0.80), while GHOST#4 showed the lowest mean (1.74, SD = 0.81). Most items demonstrated relatively normal distributions with slight positive skewness. The total GQ score averaged 17.02 (SD = 4.47) with good internal consistency (α = 0.88). Test–retest reliability was excellent (ICC = 0.92) over a two-week interval (Table 1).

#### 3.2.2. RG-C Results

The Role of Victim subscale showed the highest mean score (20.93, SD = 3.37), followed by Role of Aggressor (20.48, SD = 3.26). The Emotional Impact subscale had the lowest mean (19.64, SD = 2.86). All subscales demonstrated good to excellent internal consistency (α = 0.90, 0.88, and 0.83, respectively). Distribution patterns were relatively normal across all three subscales (Table 1).

### 3.3. Psychometric Results

#### 3.3.1. CFA Results

The single-factor model for the GH demonstrated acceptable to excellent fit across multiple indices. All eight GHOST items loaded significantly on the single ghosting factor (*p* < 0.001). Standardized factor loadings ranged from 0.464 (GHOST#1) to 0.688 (GHOST#8). GHOST#8 showed the strongest loading (0.688), while GHOST#1 had the weakest (0.464). All loadings exceeded the 0.40 threshold for acceptable factor representation. Comparative fit indices indicated excellent model fit. CFI (0.963), TLI (0.948), IFI (0.964), and RNI (0.963) all exceeded the 0.950 threshold for excellent fit. NFI reached acceptable levels (0.927 > 0.90), while RFI approached the threshold (0.898). SRMR (0.037) and RMSEA (0.058) both indicated good to acceptable fit (<0.08). Standardized residual variances ranged from 0.527 to 0.785, indicating adequate item reliability. The model showed sufficient convergent validity with AVE around 0.50. Information criteria (AIC = 5039.083, BIC = 5125.798) supported model parsimony. The single-factor structure adequately represented the ghosting construct in this French sample (Table 2).

Factor invariance testing confirmed stable measurement properties across country, sex and relationship status. The main invariance aimed to examine whether the French GHOST functioned equivalently across country groups; therefore, we tested measurement invariance between Tunisian (administrative French) and Bahraini (third language French) participants. Sequential confirmatory factor analyses supported configural invariance (χ^2^(40) = 63.57, *p* = 0.01; CFI = 0.95; RMSEA = 0.07), metric invariance (χ^2^(47) = 67.12, *p* = 0.03; CFI = 0.96; RMSEA = 0.06), and scalar invariance (χ^2^(62) = 80.27, *p* = 0.06; CFI = 0.96; RMSEA = 0.05). Changes in CFI between nested models did not exceed 0.01 (ΔCFI ≤ 0.01), satisfying established criteria for measurement equivalence.

To ensure the French GHOST measured ghosting experiences equivalently for men and women, we tested measurement invariance across gender groups (females vs. males). Sequential confirmatory factor analyses supported metric invariance (χ^2^(47) = 71.46, *p* = 0.01; CFI = 0.95; RMSEA = 0.06), scalar invariance (χ^2^(54) = 76.00, *p* = 0.03; CFI = 0.96; RMSEA = 0.05), and strict invariance (χ^2^(62) = 81.81, *p* = 0.05; CFI = 0.96; RMSEA = 0.05). Changes in CFI between nested models did not exceed 0.01 (ΔCFI ≤ 0.01), meeting established criteria for measurement equivalence. The goodness of fit index was excellent for both scalar (GFI = 0.99) and strict (GFI = 0.99) models.

To also verify that the French GHOST functioned equivalently regardless of recent romantic relationship experience, we tested measurement invariance across relationship status groups (currently/recently in relationship vs. not in relationship in past six months). Sequential confirmatory factor analyses supported metric invariance (χ^2^(47) = 69.92, *p* = 0.02; CFI = 0.95; RMSEA = 0.06), scalar invariance (χ^2^(54) = 81.34, *p* < 0.01; CFI = 0.95; RMSEA = 0.06), and strict invariance (χ^2^(62) = 90.70, *p* = 0.01; CFI = 0.94; RMSEA = 0.06). Changes in CFI between nested models remained within acceptable limits (ΔCFI ≤ 0.01), confirming measurement equivalence. The goodness of fit indices were excellent for both scalar (GFI = 0.99) and strict (GFI = 0.99) models.

#### 3.3.2. IRT Results

The complete IRT analysis of GHOST is shown in Table 3. 

The polytomous Rasch model analysis revealed differential item functioning across the eight GHOST items. Person reliability was good (0.83), indicating consistent measurement across respondents. Item difficulty measures ranged from −2.16 (GHOST#1) to −0.10 (GHOST#4). GHOST#1 was the easiest item to endorse, while GHOST#4 represented the most difficult ghosting behavior to acknowledge. GHOST#2 (−1.76) and GHOST#6 (−1.57) were also relatively easy to endorse. Standard errors ranged from 0.08 to 0.10, indicating precise parameter estimation. The delta-tau parameterization showed varying response category thresholds across items. Initial thresholds (category 1) were consistently negative, ranging from −28.40 to −34.10. Higher category thresholds showed more variable patterns. GHOST#1 demonstrated the highest final threshold (13.30), while GHOST#3 showed the lowest (9.49). All items utilized the full five-point response scale effectively. Threshold progression was generally monotonic, indicating proper category ordering. Some items showed compressed middle categories, suggesting potential response scale optimization. The marginal maximum likelihood estimation provided stable parameter estimates across all items. The Rasch model analysis confirmed unidimensional measurement of ghosting behaviors. Item hierarchy revealed that admitting to more severe ghosting behaviors (GHOST#4) was harder than acknowledging milder forms (GHOST#1). This pattern supports the construct validity of the ghosting continuum (Table 3). 

#### 3.3.3. Correlation Between GH and RG-C

Strong positive correlations were found between the GQ and all RG-C subscales. All correlations were statistically significant at *p* < 0.001 (Table 4).

The GQ showed the strongest correlation with the Role of Aggressor subscale (r = 0.899), indicating substantial overlap between general ghosting behaviors and perpetrator experiences. The correlation with Role of Victim was also strong (r = 0.829), suggesting that ghosting perpetrators often experience being ghosted themselves (Table 4).

The Role of Aggressor and Role of Victim subscales were highly correlated (r = 0.763), indicating that individuals who ghost others frequently experience being ghosted. Both aggressor and victim roles showed moderate correlations with Emotional Impact (r = 0.593 and r = 0.574, respectively) (Table 4).

The Emotional Impact subscale showed the weakest correlation with the GQ (r = 0.632), though still indicating a strong relationship. This suggests that while ghosting behaviors relate to emotional consequences, the relationship is less direct than with behavioral roles (Table 4).

The strong correlations between measures support convergent validity of both instruments. The pattern suggests that ghosting represents a multifaceted construct encompassing both behavioral and emotional dimensions. The high intercorrelations indicate these measures assess related but distinct aspects of romantic ghosting experiences.

## 4. Discussion

The French GHOST demonstrates robust psychometric properties as a reliable and valid instrument for measuring ghosting experiences. Its excellent internal consistency (α = 0.88) and test–retest reliability (ICC = 0.92) establish strong measurement stability. The CFA revealed excellent model fit (CFI = 0.963, TLI = 0.948, RMSEA = 0.058), supporting the unidimensional structure consistent with the original English version. The substantial correlation with the RG-C Role of Aggressor subscale (r = 0.899) provides compelling evidence of convergent validity. Item response theory analysis confirmed proper functioning across all response categories and revealed a meaningful item hierarchy from mild to severe ghosting behaviors. Factor invariance testing across country, sex and relationship status demonstrated stable measurement properties, affirming the instrument’s cross-cultural consistency and broad applicability in French-speaking populations. Specifically, country findings demonstrated that the French GHOST measures the same construct equivalently across participants with different relationships to the French language (administrative/educational language in Tunisia vs. third language in Bahrain), supporting the instrument’s cross-cultural validity and justifying the combination of samples for subsequent analyses.

The successful validation of the French GHOST contributes to a growing body of evidence supporting the cross-cultural applicability of this instrument for measuring ghosting experiences. This comparative analysis examines the psychometric properties of the French GHOST in relation to previously validated versions across multiple languages and cultural contexts (Husain et al., 2024a, 2024b; Özalp et al., 2025), providing insights into the universal nature of ghosting behaviors and the methodological evolution of validation research.

The reliability coefficients across validated GHOST versions demonstrate consistently strong internal consistency, with notable variations that warrant examination. The French adaptation achieved a Cronbach’s alpha of 0.88, positioning it within the range of excellent reliability alongside the Urdu version (α = 0.913) and Arabic version (α = 0.87). The Turkish adaptations maintained high reliability with alphas ranging from 0.876 to 0.879 across different validation phases. Notably, the original English version reported the lowest reliability coefficient at α = 0.74, though this remains within acceptable psychometric standards.

This pattern suggests that the translation and cultural adaptation process may contribute to enhanced internal consistency, potentially through improved item clarity or cultural relevance. However, methodological differences between validation studies, including sample characteristics and data collection procedures, may also account for these variations.

The CFA results across all validated versions consistently support a unidimensional structure, providing robust evidence for the conceptual equivalence of the GHOST construct across diverse linguistic and cultural contexts. The French version demonstrated excellent model fit indices (CFI = 0.963, TLI = 0.948, RMSEA = 0.058, SRMR = 0.037), surpassing the original English version (CFI = 0.92, TLI = 0.90, RMSEA = 0.07, SRMR = 0.04) (Jahrami et al., 2023) and closely aligning with the superior fit statistics of the Urdu adaptation (CFI = 0.991, TLI = 0.987, RMSEA = 0.045, SRMR = 0.018) (Husain et al., 2024a).

The consistent single-factor structure across culturally diverse populations supports the theoretical framework of ghosting as a unified construct while demonstrating the instrument’s cross-cultural validity. Furthermore, measurement invariance testing conducted for the French GHOST confirmed stable measurement properties across demographic variables, consistent with findings from other language adaptations.

Test–retest reliability data reveal consistently high temporal stability across available validations. The French GHOST demonstrated exceptional stability with an ICC of 0.92 over a two-week interval, comparable to the Urdu version (ICC = 0.960) and the Arabic version (ICC = 0.89). These findings provide strong evidence for the instrument’s reliability over time, though the absence of comparable data for the original English version limits comprehensive comparison.

The high temporal stability coefficients across different cultural contexts suggest that ghosting experiences, as measured by the GHOST, represent stable individual differences rather than transient behavioral patterns, supporting the instrument’s utility for longitudinal research applications.

A notable trend across successive GHOST validations is the increasing sophistication of psychometric evaluation procedures. While the original English validation established foundational psychometric properties, subsequent adaptations have incorporated advanced analytical techniques including IRT analysis, measurement invariance testing, and comprehensive convergent validity assessments.

The French, Arabic, and Turkish validations all employed IRT analysis, revealing meaningful item hierarchies that progress from mild to severe ghosting behaviors. This analytical approach provides deeper insights into the construct’s dimensional properties and enhances understanding of the ghosting continuum. The Turkish validations additionally examined relationships with personality traits and emotional variables, extending the nomological network beyond the scope of earlier studies.

The French GHOST’s convergent validity profile, characterized by strong correlations with the Romantic Ghosting Scale subscales, particularly the “Role of Aggressor” dimension (r = 0.899), parallels patterns observed in other validations. This consistent finding across cultural contexts suggests that individuals who experience ghosting frequently engage in ghosting behaviors themselves, supporting a bidirectional conceptualization of the phenomenon.

The Urdu validation’s high correlation with the original English questionnaire (r = 0.947) and the Turkish version’s significant associations with personality dimensions provide complementary evidence for the instrument’s convergent validity across diverse theoretical frameworks and cultural contexts.

The consistent psychometric performance across five language adaptations spanning individualistic and collectivistic cultural contexts provides compelling evidence for the cross-cultural generalizability of the GHOST construct. The instrument’s effectiveness across populations varying in religious orientation, traditional values, and social structures suggests that ghosting behaviors transcend cultural boundaries.

However, all validation studies share common methodological limitations, particularly reliance on convenience sampling and predominant recruitment from university populations. This sampling approach may limit generalizability to broader demographic groups and introduce systematic biases related to digital literacy and relationship experience. The consistent use of convenience samples, while facilitating cross-study comparisons, restricts the external validity of findings and necessitates cautious interpretation of results across different age groups and socioeconomic strata.

The demonstrated cross-cultural validity of the GHOST establishes it as a valuable instrument for international research collaboration and comparative studies of ghosting phenomena. The instrument’s brief administration time (under five minutes) and robust psychometric properties make it suitable for large-scale epidemiological studies and clinical screening applications.

The measurement invariance findings across demographic variables support the instrument’s utility for examining group differences within populations, while the consistent factor structure enables meaningful cross-cultural comparisons. These properties are particularly valuable for researchers investigating the psychological correlates and consequences of ghosting across diverse cultural contexts.

The strong correlations between the French GHOST and the RG-C subscales (r = 0.899 with the Role of Aggressor; r = 0.829 with the Role of Victim) may suggest redundancy between the instruments; however, several critical distinctions justify the independent utility of the French GHOST and underscore the necessity for a French-language ghosting measure. First, it is important to note that the RG-C was administered in English in the current study, thereby indicating that no validated French ghosting instrument existed prior to this research. The RG-C was developed and validated exclusively in Spanish for Colombian populations (Herrera-López et al., 2024), and to our knowledge, no validated French translation has been published. Utilizing the English version for convergent validity purposes does not address the essential requirement for a French-language instrument accessible to Francophone researchers and clinicians. Second, the instruments differ significantly in their scope and intended application. The RG-C is explicitly designed to assess ghosting solely within romantic relationships, with all items contextualized to romantic partners and dating scenarios. Conversely, the GHOST is intentionally designed as a context-general measure applicable across various relationship types-romantic, platonic, familial, professional, and therapeutic (Jahrami et al., 2023). This broader scope reflects empirical evidence indicating that ghosting occurs across multiple relational domains (Astleitner et al., 2023; Farber et al., 2022; Wood et al., 2023), allowing researchers to examine ghosting experiences comprehensively rather than confining assessments to romantic contexts. For studies investigating ghosting in workplace settings, friendships, or family relationships, the RG-C would be inappropriate, while the GHOST remains relevant. Third, the instruments vary in length and assessment burden. The GHOST consists of 8 items and takes approximately five minutes to complete, whereas the RG-C contains 15 items, with a dual-response format for 10 items (requiring participants to rate both their own behavior and their partner’s behavior for each item), resulting in effectively 25 response points and a longer administration time. For large-scale epidemiological studies, clinical screening, or research protocols involving multiple measures, the GHOST’s brevity provides practical advantages without compromising psychometric quality. Fourth, the differential correlation pattern observed in Table 4 indicates that these instruments capture related but distinct aspects of ghosting. The GHOST demonstrated its strongest correlation with the RG-C Role of Aggressor subscale (r = 0.899), which may initially appear counterintuitive given that the GHOST primarily assesses experiences of being ghosted (victim experiences). However, this pattern aligns with bidirectional ghosting theory-individuals who experience being ghosted are also more likely to ghost others (Powell et al., 2021; Koessler et al., 2019). The somewhat lower correlation with Emotional Impact (r = 0.632) suggests that the GHOST captures behavioral experiences of ghosting more directly than emotional consequences, indicating that these instruments assess partially overlapping but non-identical constructs. If the instruments were redundant, uniformly high correlations across all RG-C subscales approaching r > 0.90 would be expected. Finally, the theoretical frameworks differ. The GHOST was developed based on communication theory and ostracism models (Williams, 2007, 2009; Jahrami et al., 2023), emphasizing communication disruption, ambiguity, and relational barriers as core dimensions. In contrast, the RG-C was developed within a relational violence framework specific to romantic relationships (Herrera-López et al., 2024), conceptualizing ghosting as a form of psychological aggression between romantic partners. These distinct theoretical foundations result in different emphases on item content-the GHOST focuses on communication patterns and experiences of exclusion, while the RG-C emphasizes behavioral tactics and emotional consequences within romantic dyads.

### Limitations

The convenience sample may not fully represent all French-speaking populations. Online recruitment risks self-selection bias, potentially overrepresenting digitally active individuals. The study also focused on adults, possibly missing adolescent ghosting dynamics. Furthermore, the current study did not establish normative data or clinical cut-off scores for the French GHOST. While our sample offers preliminary descriptive statistics (M = 17.02, SD = 4.47 for total scores), future research utilizing larger and more representative samples should aim to develop age- and context-specific norms to enhance clinical interpretation and screening applications. Conducting Receiver Operating Characteristic (ROC) curve analyses that compare GHOST scores with clinical diagnoses or measures of functional impairment would be beneficial for determining clinically meaningful thresholds.

## 5. Conclusions

The French GHOST stands as a psychometrically sound and culturally appropriate instrument, providing a vital tool for researchers and clinicians to explore the psychological impacts of ghosting and to inform culturally sensitive interventions within French-speaking communities. Future research directions include establishing normative data and clinical cut-off scores for French-speaking populations, examining the instrument’s sensitivity to change in longitudinal studies, and investigating its utility in clinical settings for identifying individuals experiencing psychological distress related to ghosting.

## Figures and Tables

**Table 1:** Descriptive Statistics of the Participants (N = 274).

Variable	Mean	SD	Minimum	Maximum
Age (in years)	23.56	4.07	18	49
GHOST#1	2.13	0.70	1	4
GHOST#2	2.52	0.80	1	5
GHOST#3	2.28	0.88	1	5
GHOST#4	1.74	0.81	1	5
GHOST#5	1.87	0.83	1	5
GHOST#6	2.42	0.91	1	5
GHOST#7	2.05	0.94	1	5
GHOST#8	2.02	0.92	1	5
GQ	17.02	4.47	8	35
RA	20.48	3.26	12	31
RV	20.93	3.37	11	31
EI	19.64	2.86	11	27

Notes: GHOST#1 to GHOST #8 corresponds to the items of the Ghosting Questionnaire (GQ)/GHOST. RA, RV, and EI represent the three subscales of the Romantic Ghosting Scale (RG-C): Role of Aggressor, Role of Victim, and Emotional Impact, respectively. Internal consistency was assessed using Cronbach’s alpha (α), with values of 0.88 for GHOST, 0.90 for RA, 0.88 for RV, and 0.83 for EI. McDonald’s omega (ω) for the GHOST was 0.88. The ICC, calculated in a subsample of 50 participants who completed the GH twice with a two-week interval, was 0.92.

**Table 2:** Confirmatory factor analysis of the Ghosting Questionnaire (GQ)/GHOST (N = 274).

Factor	Indicator	Standardized Factor Loadings				Standardized Residual Variance			
		Loading	Std. Error	z-Value	*p*	Loading	Std. Error	z-Value	*p*
GH	GHOST#1	0.464	0.044	7.359	<0.001	0.785	0.035	10.979	<0.001
	GHOST#2	0.526	0.050	8.443	<0.001	0.724	0.044	10.636	<0.001
	GHOST#3	0.550	0.054	8.901	<0.001	0.697	0.052	10.486	<0.001
	GHOST#4	0.667	0.048	11.315	<0.001	0.556	0.038	9.558	<0.001
	GHOST#5	0.573	0.051	9.409	<0.001	0.671	0.045	10.412	<0.001
	GHOST#6	0.606	0.055	10.055	<0.001	0.633	0.052	10.167	<0.001
	GHOST#7	0.628	0.056	10.472	<0.001	0.605	0.053	9.906	<0.001
	GHOST#8	0.688	0.054	11.73	<0.001	0.527	0.048	9.238	<0.001

Notes: GH = Ghosting Questionnaire/GHOST. Estimator Maximum likelihood estimation (MLE). Factor Model: χ^2^ = 38.715, df = 20, *p* = 0.007. Baseline Model: χ^2^ = 533.352, df = 28. CFI = 0.963 (>0.95 indicates excellent fit). TLI/NNFI = 0.948 (>0.95 indicates excellent fit). IFI = 0.964 (>0.95 indicates excellent fit). RNI = 0.963 (>0.95 indicates excellent fit). NFI = 0.927 (>0.90 indicates acceptable fit). RFI = 0.898 (approaching 0.90 threshold). SRMR = 0.037 (<0.08 indicates good fit). RMSEA = 0.058 (<0.08 indicates acceptable fit). AIC: 5039.083. BIC: 5125.798. SSABIC: 5049.699. Log-likelihood: −2495.54. GFI: 0.994 (excellent fit, >0.95). MFI: 0.966 (excellent fit). ECVI: 0.316 (lower values indicate better expected replication). AVE around 0.50 indicates sufficient convergent validity. Factor invariance testing across country, sex and relationship status demonstrated stable measurement properties.

**Table 3:** Item response analysis of the Ghosting Questionnaire (GQ)/GHOST (N = 274).

Item Statistics of the Rating Scale Model			Delta-Tau Parameterization of the Partial Credit Model				
Variable	Measure	Std. Error	1	2	3	4	5
GHOST#1	−2.16	0.09	−28.40	6.77	9.63	12.05	13.30
GHOST#2	−1.76	0.08	−32.50	4.52	7.65	9.37	11.00
GHOST#3	−1.30	0.09	−30.30	4.92	7.15	8.78	9.49
GHOST#4	−0.10	0.10	−33.00	6.26	7.44	9.81	9.52
GHOST#5	−0.42	0.09	−34.10	6.05	8.38	8.67	10.97
GHOST#6	−1.57	0.08	−30.50	5.05	6.83	8.76	9.92
GHOST#7	−0.83	0.09	−31.40	5.81	7.79	8.94	8.91
GHOST#8	−0.75	0.09	−31.40	5.72	7.20	8.52	9.97

Notes: Estimator Polytomous Rasch Model using Marginal Maximum likelihood Estimation (MMLE). Person reliability was 0.83.

**Table 4:** Correlations between Ghosting Questionnaire (GHOST) and the Romantic Ghosting Scale (RG-C).

Variable	GQ	RA	RV	EI
GQ	—			
RA	0.899 *	—		
RV	0.829 *	0.763 *	—	
EI	0.632 *	0.593 *	0.574 *	—

Notes: Pearson product-moment correlation coefficient. * indicates significance at 0.001. GQ = Ghosting Questionnaire/GHOST. RA, RV, EI are the three subscales of Romantic Ghosting Scale (RG-C).

## Data Availability

Data associated with this paper can be produced upon request from the corresponding author.

## References

[B1-ejihpe-16-00015] Adler N. J., Graham J. L. (2009). Business negotations: Canadians are not just like Americans. Canadian Journal of Administrative Sciences/Revue Canadienne des Sciences de l’Administration.

[B2-ejihpe-16-00015] Astleitner H., Bains A., Hörmann S. (2023). The effects of personality and social media experiences on mental health: Examining the mediating role of fear of missing out, ghosting, and vaguebooking. Computers in Human Behavior.

[B3-ejihpe-16-00015] Baumeister R. F., Leary M. R. (1995). The need to belong: Desire for interpersonal attachments as a fundamental human motivation. Psychological Bulletin.

[B4-ejihpe-16-00015] Beaton D. E., Bombardier C., Guillemin F., Ferraz M. B. (2000). Guidelines for the process of cross-cultural adaptation of self-report measures. Spine.

[B5-ejihpe-16-00015] Biolcati R., Pupi V., Mancini G. (2021). Cyber dating abuse and ghosting behaviours: Personality and gender roles in romantic relationships. Current Issues in Personality Psychology.

[B6-ejihpe-16-00015] Boss P. (2016). The context and process of theory development: The story of ambiguous loss. Journal of Family Theory & Review.

[B7-ejihpe-16-00015] Chalmers R. P. (2012). mirt: A multidimensional item response theory package for the R environment. Journal of Statistical Software.

[B8-ejihpe-16-00015] Daraj L. R., Buhejji M. R., Perlmutter G., Jahrami H., Seeman M. V. (2023). Ghosting: Abandonment in the digital era. Encyclopedia.

[B9-ejihpe-16-00015] Downey G., Feldman S. I. (1996). Implications of rejection sensitivity for intimate relationships. Journal of Personality and Social Psychology.

[B10-ejihpe-16-00015] Farber B. A., Hubbard E., Ort D. (2022). Patients’ experiences of being “ghosted” by their psychotherapists. Psychotherapy.

[B11-ejihpe-16-00015] Fewou Ngouloure J. P. (2023). La Francophonie au cœur de l’écosystème numérique: Enjeux et ébauche de méthode. Nouveaux Cahiers de Marge.

[B12-ejihpe-16-00015] Forrai M., Koban K., Matthes J. (2023). Short-sighted ghosts. Psychological antecedents and consequences of ghosting others within emerging adults’ romantic relationships and friendships. Telematics and Informatics.

[B13-ejihpe-16-00015] Freedman G., Hales A. H., Powell D. N., Le B., Williams K. D. (2022). The role of gender and safety concerns in romantic rejection decisions. Journal of Experimental Social Psychology.

[B14-ejihpe-16-00015] Freedman G., Powell D. N. (2024). Ghosting: A common but unpopular rejection strategy. Social and Personality Psychology Compass.

[B15-ejihpe-16-00015] Freedman G., Powell D. N., Le B., Williams K. D. (2019). Ghosting and destiny: Implicit theories of relationships predict beliefs about ghosting. Journal of Social and Personal Relationships.

[B16-ejihpe-16-00015] Freedman G., Powell D. N., Le B., Williams K. D. (2024). Emotional experiences of ghosting. The Journal of Social Psychology.

[B17-ejihpe-16-00015] Gregoire J. (2018). ITC guidelines for translating and adapting tests (second edition). International Journal of Testing.

[B18-ejihpe-16-00015] Herrera-López M., Coral-Lagos A., Enriquez-Rosero M., Herrera-Solarte L. (2024). Propiedades psicométricas de la Escala de Ghosting Romántico RG-C: Un estudio instrumental en una muestra colombiana. Psychology, Society & Education.

[B19-ejihpe-16-00015] Husain W., Ammar A., Trabelsi K., AlSaleh A., Jahrami H. (2025). Avoid and rule: Selective sociality scale for understanding introverted personality in a digitally socialized world. European Journal of Investigation in Health, Psychology and Education.

[B20-ejihpe-16-00015] Husain W., Sadiqa A., Zahid E., Idrees F., Ammar A., Saif Z., Trabelsi K., Pandi-Perumal S. R., Seeman M. V., Jahrami H. (2024a). The translation and preliminary psychometric validation of the ghosting questionnaire in Urdu. European Journal of Investigation in Health, Psychology and Education.

[B21-ejihpe-16-00015] Husain W., Salem A. A. M. S., Ammar A., Trabelsi K., Ghazzawi H., Saif Z., Helmy M., Pandi-Perumal S. R., Seeman M. V., Pakpour A. H., Jahrami H. (2024b). Translating and validating the ghosting questionnaire into Arabic: Results from classical test theory and item response theory analyses. BMC Psychology.

[B22-ejihpe-16-00015] Jahrami H., Saif Z., Chen W., Helmy M., Ghazzawi H., Trabelsi K., Natan Pires G., Bragazzi N. L., Pandi-Perumal S. R., Seeman M. V. (2023). Development and validation of a questionnaire (GHOST) to assess sudden, unexplained communication exclusion or “ghosting”. Heliyon.

[B23-ejihpe-16-00015] Jonason P. K., Kaźmierczak I., Campos A. C., Davis M. D. (2021). Leaving without a word: Ghosting and the Dark Triad traits. Acta Psychologica.

[B24-ejihpe-16-00015] Jorgensen T. D., Pornprasertmanit S., Schoemann A. M., Rosseel Y., Miller P., Quick C., Garnier-Villarreal M., Selig J., Boulton A., Preacher K. (2025). semTools: Useful tools for structural equation modeling version 0.5-7.

[B25-ejihpe-16-00015] Kay C., Courtice E. L. (2022). An empirical, accessible definition of “ghosting” as a relationship dissolution method. Personal Relationships.

[B26-ejihpe-16-00015] Koessler R. B., Kohut T., Campbell L., Vazire S., Chopik W. (2019). When your boo becomes a ghost: The association between breakup strategy and breakup role in experiences of relationship dissolution. Psychology Collabra.

[B27-ejihpe-16-00015] Konings F., Sumter S., Vandenbosch L. (2023). It’s not you, it’s me: Experiences with ghosting on mobile dating applications and Belgian emerging adults’ self-esteem. Sexuality & Culture.

[B28-ejihpe-16-00015] LeFebvre L. E., Allen M., Rasner R. D., Garstad S., Wilms A., Parrish C. (2019). Ghosting in emerging adults’ romantic relationships: The digital dissolution disappearance strategy. Imagination, Cognition and Personality.

[B29-ejihpe-16-00015] LeFebvre L. E., Fan X. (2020). Ghosted?: Navigating strategies for reducing uncertainty and implications surrounding ambiguous loss. Personal Relationships.

[B30-ejihpe-16-00015] Mair P., Hatzinger R., Maier M. J. (2025). eRm: Extended Rasch modeling. R package version 1.0-10.

[B31-ejihpe-16-00015] Martin E. (2011). Multilingualism and Web advertising: Addressing French-speaking consumers. Journal of Multilingual and Multicultural Development.

[B32-ejihpe-16-00015] Meikle G. (2024). Social media: The convergence of public and personal communication.

[B33-ejihpe-16-00015] Navarro R., Larrañaga E., Yubero S., Víllora B. (2020). Psychological correlates of ghosting and breadcrumbing experiences: A preliminary study among adults. International Journal of Environmental Research and Public Health.

[B34-ejihpe-16-00015] Özalp M., Husain W., Yaman K. G., Ammar A., Trabelsi K., Pandi-Perumal S. R., Jahrami H. (2025). Turkish adaptation of the ghosting questionnaire and its impact on relationship satisfaction: Serial mediation effects of negative affect and loneliness. European Journal of Investigation in Health, Psychology and Education.

[B35-ejihpe-16-00015] Pancani L., Aureli N., Riva P. (2022). Relationship dissolution strategies: Comparing the psychological consequences of ghosting, orbiting, and rejection. Cyberpsychology: Journal of Psychosocial Research on Cyberspace.

[B36-ejihpe-16-00015] Pancani L., Mazzoni D., Aureli N., Riva P. (2021). Ghosting and orbiting: An analysis of victims’ experiences. Journal of Social and Personal Relationships.

[B37-ejihpe-16-00015] Powell D. N., Freedman G., Le B., Williams K. D. (2022). Exploring individuals’ descriptive and injunctive norms of ghosting. Cyberpsychology: Journal of Psychosocial Research on Cyberspace.

[B38-ejihpe-16-00015] Powell D. N., Freedman G., Williams K. D., Le B., Green H. (2021). A multi-study examination of attachment and implicit theories of relationships in ghosting experiences. Journal of Social and Personal Relationships.

[B39-ejihpe-16-00015] Revelle W. (2025). psych: Procedures for psychological, psychometric, and personality research. R package version, 2.5.6.

[B40-ejihpe-16-00015] Rosseel Y. (2012). lavaan: An R package for structural equation modeling. Journal of Statistical Software.

[B41-ejihpe-16-00015] Shannon C. E. (1949). Communication theory of secrecy systems. Bell System Technical Journal.

[B42-ejihpe-16-00015] Thomas J. O., Dubar R. T. (2021). Disappearing in the age of hypervisibility: Definition, context, and perceived psychological consequences of social media ghosting. Psychology of Popular Media.

[B43-ejihpe-16-00015] Timmermans E., Hermans A.-M., Opree S. J. (2020). Gone with the wind: Exploring mobile daters’ ghosting experiences. Journal of Social and Personal Relationships.

[B44-ejihpe-16-00015] von Elm E., Altman D. G., Egger M., Pocock S. J., Gøtzsche P. C., Vandenbroucke J. P. (2008). The strengthening the reporting of observational studies in epidemiology (STROBE) statement: Guidelines for reporting observational studies. Journal of Clinical Epidemiology.

[B45-ejihpe-16-00015] Wei T., Simko V., Levy M., Xie Y., Jin Y., Zemla J. (2024). R package. Corrplot: Visualization of a correlation matrix version 0.95.

[B46-ejihpe-16-00015] Williams K. D. (2007). Ostracism: The kiss of social death. Social and Personality Psychology Compass.

[B47-ejihpe-16-00015] Williams K. D. (2009). Ostracism: A temporal need-threat model. Advances in Experimental Social Psychology.

[B48-ejihpe-16-00015] Wood N. R., Leckfor C. M., Wicks S. G., Hales A. H. (2023). Ghosting from the workplace: The impact of feedback (or lack thereof) on applicants’ psychological needs satisfaction. Routledge Open Research.

[B49-ejihpe-16-00015] Wu K., Bamishigbin O. (2023). When silence speaks louder than words: Exploring the experiences and attitudes of ghosters. Personal Relationships.

